# Immune dysregulation and endothelial dysfunction associate with a pro-thrombotic profile in Long COVID

**DOI:** 10.3389/fimmu.2025.1613195

**Published:** 2025-10-16

**Authors:** Alicia Simón-Rueda, Clara Sánchez-Menéndez, Guiomar Casado, Daniel Fuertes, María Aránzazu Murciano-Antón, Elena Mateos, Susana Domínguez-Mateos, Francisco Pozo, Javier García-Pérez, Mayte Pérez-Olmeda, Miguel Cervero, Marta Massanella, Gemma Moncunill, Montserrat Torres, Mayte Coiras

**Affiliations:** ^1^ Immunopathology and Viral Reservoir Unit, National Center of Microbiology, Instituto de Salud Carlos III, Madrid, Spain; ^2^ PhD Program in Biomedical Sciences and Public Health, Universidad Nacional de Educación a Distancia (UNED), Madrid, Spain; ^3^ Biomedical Research Center Network in Infectious Diseases (CIBERINFEC), Instituto de Salud Carlos III, Madrid, Spain; ^4^ Infectious Diseases Unit, Internal Medicine Service, Hospital Clínico San Carlos, Madrid, Spain; ^5^ School of Telecommunications Engineering, Universidad Politécnica de Madrid, Madrid, Spain; ^6^ Family Medicine Service, Primary Healthcare Center Doctor Pedro Laín Entralgo, Madrid, Spain; ^7^ Family Medicine Service, Primary Healthcare Center Arroyomolinos, Madrid, Spain; ^8^ Respiratory Viruses Unit, National Center of Microbiology, Instituto de Salud Carlos III, Madrid, Spain; ^9^ Biomedical Research Center Network in Epidemiology and Public Health (CIBERESP), Instituto de Salud Carlos III, Madrid, Spain; ^10^ AIDS Immunopathology Unit, National Center of Microbiology, Instituto de Salud Carlos III, Madrid, Spain; ^11^ Serology Service, National Center of Microbiology, Instituto de Salud Carlos III, Madrid, Spain; ^12^ School of Medicine, Universidad Alfonso X El Sabio, Madrid, Spain; ^13^ IrsiCaixa AIDS Research Institute, Germans Trias i Pujol Research Institute (IGTP), Can Ruti Campus, Barcelona, Spain; ^14^ REICOP, Spanish Network for Persistent COVID Research, Madrid, Spain; ^15^ Instituto de Salud Global de Barcelona (ISGLOBAL), Barcelona, Spain; ^16^ Facultat de Medicina i Ciències de la Salut, Universitat de Barcelona (UB), Barcelona, Spain

**Keywords:** Long COVID, immunity, COVID-19 vaccines, endothelium, blood coagulation disorders, biomarkers

## Abstract

**Introduction:**

Long COVID (LC) affects approximately 10% of individuals post-SARS-CoV-2 infection, with symptoms persisting beyond 12 weeks. The underlying mechanisms remain unclear, and current models often focus on pre-existing comorbidities.

**Methods:**

This cohort study aimed to identify robust biomarkers and clarify LC pathogenesis through a comprehensive analysis performed in 32 LC individuals 26 months post-infection compared with 35 fully recovered individuals recruited between March and July 2022. Blood and fecal samples were collected, and multiple parameters associated with immune dysfunction, endothelial damage, bacterial translocation, and coagulation alterations, alongside signs of viral persistence and sociodemographic and clinical features, were analyzed.

**Results:**

Although viral RNA was undetected on blood or stool, elevated plasma IgG against the nucleocapsid may indicate frequent reinfections, greater infection severity, or delayed immune normalization. Increased levels of prothrombin, thrombin, fibrinogen, sEPCR, and CRP pointed to persistent endothelial dysfunction and coagulation imbalance. Lower levels of the bactericidal protein REG3A suggest potential disruptions in mucosal immune response. We found no major differences in traditional comorbidities, highlighting that LC may stem from distinct pathogenic mechanisms beyond pre-existing conditions. Importantly, our study revealed impaired humoral immunity and identified an association between vaccine heterogeneity and increased LC risk, emphasizing the relevance of consistent vaccination strategies. A Random Forest model using the measured biomarkers achieved 100% accuracy in classifying LC individuals, reinforcing their diagnostic potential.

**Discussion:**

These findings support a multifactorial model of LC involving immune dysregulation and persistent endothelial damage that led to coagulation abnormalities and a pro-thrombotic profile, supporting that LC is more closely related to a sustained, uncontrolled inflammatory response rather than immunodeficiency, and underscoring the value of multidimensional biomarker profiling for guiding clinical management and prevention strategies.

## 1 Introduction

The COVID-19 pandemic, caused by SARS-CoV-2, has profoundly impacted global health systems and societies. Since the World Health Organization (WHO) declared it a pandemic in 2020, over 770 million confirmed infections and 7 million deaths have been reported, though the actual numbers are likely higher (1).

Effective vaccination campaigns reduced viral spread and mitigated severe infections, but Long COVID (LC) remains a significant consequence. Also known as Post-acute COVID-19 Syndrome (PACS) or Post-COVID-19 Condition (PCC), LC is characterized by symptoms persisting beyond 12 weeks after infection, affecting 10–20% of individuals (2). Symptoms vary widely, including fatigue, breathlessness, myalgia, palpitations, gastrointestinal issues, cognitive impairment, and mental health disorders such as anxiety and depression (3). It is unclear whether these mental health effects result from underlying physiological conditions or impaired quality of life (4). Risk factors include female sex, older age, and comorbidities like obesity and diabetes (5). There is conflicting evidence on whether vaccination affects the risk of LC. While some reports suggest an association between LC and vaccination (Post-COVID-19 Vaccination Syndrome) (6), most evidence indicates that vaccination, whether pre- or post-infection, provides protection against LC (7).

Over 200 symptoms across multiple organ systems complicate the establishment of a diagnostic framework for this heterogeneous syndrome (8). Several hypotheses attempt to explain LC’s underlying mechanisms. One leading theory is viral persistence, where SARS-CoV-2 remains in hidden reservoirs, driving chronic inflammation and immune dysregulation (9). LC shares similarities with autoimmune conditions like fibromyalgia or chronic fatigue syndrome, which involve persistent inflammation and exaggerated immune responses (10). Evidence of viral RNA and proteins has been found in various tissues, including the respiratory and cardiovascular systems, kidneys, gastrointestinal tract, muscles, brain, and lymph nodes (2). Prolonged viral shedding in stool samples has been detected months after diagnosis, even with negative nasopharyngeal RT-PCR results (11), suggesting that viral persistence in the gut could contribute to LC-related gastrointestinal symptoms (12).

Intestinal epithelium damage and compromised barrier integrity may enable bacterial translocation, altering the gut microbiome and triggering sustained immune activation (13). Persistent endothelial dysfunction has also been observed in LC patients, evidenced by elevated markers of endothelial damage and activation (14). This dysfunction may stem from endothelial inflammation during acute infection, as SARS-CoV-2 infects endothelial cells *via* ACE2 (15). Chronic endothelial damage could underlie the systemic inflammation in LC, characterized by elevated levels of proinflammatory markers such as interleukin-6 (IL-6), C-reactive protein (CRP), and tumor necrosis factor alpha (TNFα) (16, 17). Since endothelial cells are crucial for coagulation regulation through anticoagulant factor production, including thrombin inhibitors and elements of the protein C pathway such as thrombomodulin and endothelial protein C receptor (EPCR) (18, 19), this persistent endothelial inflammation and damage may disrupt this regulation, contributing to coagulopathy in LC. In fact, while acute COVID-19 is known to cause disseminated intravascular coagulation (20), LC is associated with coagulopathies such as thrombotic endotheliitis, hyperactivated platelets, and fibrinaloid microclots, which may exacerbate the syndrome’s symptoms (17). However, although dysbiosis, persistent endothelial damage, altered coagulability, autoimmunity, or even the reactivation of latent herpesvirus such as Epstein-Barr virus (EBV) could contribute to long-lasting inflammation in people with LC (21, 22), these findings remain controversial, and their role in LC pathogenesis is not fully understood. The search for biomarkers linked to these mechanisms is critical for diagnosis, identifying therapeutic targets, and developing curative treatments.

In this cohort study, we evaluated multiple parameters associated with immune dysfunction, endothelial damage, bacterial translocation, and coagulation alterations, alongside signs of viral persistence and sociodemographic and clinical features, in a cohort of individuals with LC. Our objective was to gain insights into the mechanisms driving LC symptomatology and to identify biomarkers that may aid in its diagnosis. This knowledge may also inform the development of novel therapeutic strategies and help refine clinical guidelines for managing LC patients.

## 2 Materials and methods

### 2.1 Study subjects

Sixty-seven individuals with symptomatic COVID-19 during the first pandemic waves in Spain were recruited at the Primary Healthcare Center (PHC) Doctor Pedro Laín Entralgo (Alcorcón, Madrid, Spain) and PHC Arroyomolinos (Arroyomolinos, Madrid, Spain) in March-July 2022. Inclusion criteria required participants to be over 18 years old and have a confirmed diagnosis of acute mild COVID-19, verified either by a positive RT-qPCR test for SARS-CoV-2 in a nasopharyngeal swab or by the presence of virus-specific IgM in plasma. Participants were categorized into two groups based on the duration of symptoms’ resolution. Those who experienced at least eight clinical signs and symptoms consistent with LC more than 12 weeks after their positive diagnosis, as defined by the National Institute for Health and Care Excellence (NICE) guidelines (23), were included in the LC cohort (n=32). Conversely, individuals who fully recovered within the first four weeks post-diagnosis were included in the Recovered cohort (n=35). All participants were recruited with the collaboration of the non-profit Spanish Association of Patients with Long-COVID (Long COVID-ACTS). Cases and controls were matched on age. In order to participate, all individuals completed a comprehensive, structured questionnaire covering clinical, cognitive, and systemic symptoms. This was used to confirm eligibility for recruitment and to ensure consistent documentation of self-reported manifestations across the cohort. All participants were followed for a total of 2 years to record breakthrough infections from primary infection to blood sample collection, confirmed by SARS-CoV-2 antigens test. Sample size was calculated to achieve 80% power with an alpha level of 0.05.

### 2.2 Ethical statement

All participants provided informed written consent prior to their inclusion in the study. The study protocol (CEI PI 72_2022) was developed in accordance with the Declaration of Helsinki and received previous review and approval from the Ethics Committee of the Instituto de Salud Carlos III (IRB IORG0006384) and the Primary Care Management Commission of the Comunidad de Madrid (Spain). Participant confidentiality and anonymity were safeguarded in compliance with current Spanish and European Data Protection regulations.

### 2.3 Blood and fecal samples

Blood samples from all participants were collected using BD Vacutainer tubes containing EDTA K2 (Becton Dickinson, Franklin Lakes, NJ). These samples were promptly processed to isolate peripheral blood mononuclear cells (PBMCs) and plasma *via* Ficoll-Hypaque density gradient centrifugation (Corning Inc, Corning, NY). Fecal samples were collected in specialized containers with preservative buffer provided by Palex Medical (Barcelona, Spain). All samples were cryopreserved until analysis. Due to sample limitations, not all determinations were conducted for every sample.

### 2.4 Detection of SARS-CoV-2 RNA by RT-qPCR

The presence of SARS-CoV-2 RNA in plasma and feces was assessed using an RT-qPCR assay targeting the envelope (E) and nucleocapsid (N) genes, following the guidelines outlined in the WHO Interim Guidance for the diagnostic testing of SARS-CoV-2 (24). Viral RNA was extracted from plasma samples using the QIAamp MinElute Virus Spin Kit and from fecal samples using the QIAamp Viral RNA Kit (Qiagen Iberia, Madrid, Spain). Samples were classified as positive if the quantification cycle (Cq) value was below 42.

### 2.5 SARS-CoV-2 serology

IgG antibodies against subunit 1 (S1) from Spike (S) protein of SARS-CoV-2 were analyzed in plasma using Euroimmun Anti-SARS-CoV-2 ELISA Assay (Euroimmun, Lübeck, Germany), according to manufacturer’s instructions. Semi-quantitative results were analyzed by calculating the ratio of optical density (OD) of each sample over the calibrator. Samples were considered positive when this ratio was ≥ 0.8. In addition, IgG against the receptor binding domain (RBD), S1, subunit 2 (S2), and N proteins of SARS-CoV-2 were analyzed by chemiluminescence immunoassay (CLIA) using BioPlex 2200 SARS-CoV-2 IgG Panel (BioRad, Hercules, CA), according to the manufacturer’s instructions. Samples were considered positive as follows: S1 ≥22 binding antibody units (BAU)/mL; S2 ≥10 U/mL; RBD ≥13 BAU/mL; and N ≥24 BAU/mL.

### 2.6 SARS-CoV-2 pseudovirus neutralization assay

One single-cycle, pseudotyped SARS-CoV-2 virus (pNL4-3Δenv_SARS-CoV-2-SΔ19(G614)_Ren) was synthesized as previously described (25). Co-transfection with vector pcDNA-VSV-G was used as a control of specificity. Briefly, neutralization activity of heat-decomplemented plasma was measured by pre-incubation of pNL4-3Δenv_SARS-CoV-2-SΔ19(G614)_Ren pseudovirus (10ng p24 Gag per well) with serial dilutions of plasma (1/32 to 1/8192) for 1 hour at 37°C (25). This mixture was then added to a monolayer of Vero E6 cells and incubated for 48 hours. Vero E6 cell line (ECACC 85020206) was kindly provided by Dr Antonio Alcamí (CBM Severo Ochoa, Madrid, Spain) and it was cultured in DMEM supplemented with 10% fetal calf serum (FCS), 100U/ml penicillin/streptomycin, and 2mM L-Glutamin (Lonza, Basil, Switzerland). After incubation, cells were lysed and viral infectivity was assessed by measuring Renilla luciferase activity (Renilla Luciferase Assay, Promega, Madison, WI) in a 96-well plate luminometer Centro XS3 LB 960 with MikroWin 2010 software (Berthold Technologies, Baden-Württemberg, Germany). Titers of neutralizing IgG were calculated as 50% neutralizing dose (NT50) using non-linear regression analysis in GraphPad Prism Software v10.2.1. (GraphPad, Inc., San Diego, CA). NT50 was defined as the highest dilution of plasma that caused 50% reduction of luciferase activity, in comparison with control without plasma.

### 2.7 Herpesvirus serology

The levels of IgG against cytomegalovirus (CMV), Varicella Zoster virus (VZV), and Herpes Simplex virus 1/2 (HSV-1/2) were analyzed in plasma on the LIASON automated platform (Diasorin) with LIASON CMV IgG II, LIASON VZV IgG, and LIASON HSV-1/2 IgG CLIA assays, respectively (DiaSorin, Saluggia, Italy). The levels of IgG against EBV viral capsid antigens (VCA) were tested using LIAISON VCA IgG CLIA assay (DiaSorin). CMV IgG and VCA IgG antibody titers were calculated and expressed as U/mL; VZV IgG titers were expressed as mU/ml; and HSV-1/2 IgG titers were expressed as a ratio. Samples were considered positive or negative according to the cutoffs established by the manufacturer.

### 2.8 Analysis of herpesvirus reactivation by qPCR

Total DNA was extracted from plasma samples using QIAamp MinElute Virus Spin Kit (Qiagen Iberia). To detect the presence of DNA from EBV and CMV, used as markers of viral reactivation, DNA was amplified by qPCR with StepOnePlus Real-Time PCR System (Applied Biosystems; Thermo Fisher, Waltham, MA), using EBV R-GENE and CMV R-GENE kits (bioMérieux. Lyon, France), respectively. Data was analyzed with StepOne v2.3 Software (Thermo Fisher) and samples were considered positive when showing a calculated value of CT (Threshold cycle).

### 2.9 Detection of bacterial translocation, endothelial damage, and coagulation markers in plasma by ELISA

Plasma levels of parameters associated with bacterial translocation such as regenerating islet derived 3 alpha (REG3A) and fatty acid binding protein 2 (FABP2) were measured using Abcam ELISA Kits (Cambridge, UK), while occludin, lipopolysaccharide-binding protein (LBP), and lipopolysaccharides (LPS) were measured using Cusabio ELISA kits (Wuhan, China). Markers for coagulation parameters and endothelial damage such as thrombin, thrombomodulin, tissue-type plasminogen activator (tPA), intercellular adhesion molecule 1 (ICAM-1), and soluble EPCR (sEPCR) were measured using Abcam ELISA Kits (Cambridge, UK), while activated protein C (APC) was quantified using Abyntek Biopharma ELISA kit (Bizkaia, Spain). Data was acquired in a Tecan Sunrise Basic Microplate reader (Tecan, Männedorf, Switzerland).

### 2.10 Analysis of cytokines and coagulation factors in plasma by Luminex assay

Customized Human Magnetic Luminex Assay kit (Thermo Fisher) was used for the simultaneous detection of the following cytokines and chemokines in plasma: interleukin (IL)-1β, IL-6, IL-8, IL-10, IL-12p70, monocyte chemoattractant protein (MCP)-4, monokine induced by interferon-gamma (MIG), macrophage inflammatory protein (MIP)-3α, and myeloid progenitor inhibitor factor (MPIF). ProcartaPlex™ Human Coagulation Panel (Thermo Fisher) was used for the detection of prothrombin, Factor XI, and Factor XIII. ProcartaPlex™ Human Simplex Kits were also used for the detection of D-dimer, fibrinogen, and CRP. Data acquisition was performed with Luminex 200 Instrument System (Thermo Fisher).

### 2.11 Random forest algorithm

A Random Forest algorithm (26) was applied to evaluate the accuracy of the qualitative and quantitative variables that showed significant differences (p<0.05) between the LC and Recovered groups, aiming to identify the most important features associated with LC. To avoid bias in selecting the training, testing, and validation sets, a nested 5-fold cross-validation procedure was performed for each competing algorithm, as previously described (27, 28). The relative importance for each feature in classifying participants was determined using the Gini Variable Importance Measure (VIM) method (29).

### 2.12 Statistical analysis

Statistical analysis was conducted using GraphPad Prism v10.2.1 (GraphPad Software Inc.) and STATA 14.2 (StataCorp LLC, College Station, TX). Quantitative variables were reported as the median with interquartile range (IQR), and qualitative variables were expressed as absolute or relative frequencies. The normality of the samples was assessed using the Shapiro-Wilk test. Significance between the two cohorts was determined using the unpaired t-test or nonparametric Mann-Whitney test, based on the normality of the data. Qualitative data were compared using Fisher’s exact test or the chi-square test, as appropriate. Linear and logistic regressions were used to estimate the odds ratio (OR) and 95% confidence interval (CI) for associations between the levels of quantitative variables and the development of LC, compared to healthy donors. Logistic regression was also applied to estimate the OR and CI for qualitative variables. A p-value of < 0.05 was considered statistically significant for all comparisons.

## 3 Results

### 3.1 Sociodemographic and clinical characteristics of the participants

This single-center, cohort study recruited 67 participants. Thirty-two participants were diagnosed with LC (LC cohort). All participants in LC cohort experienced symptomatic acute COVID-19 following initial SARS-CoV-2 infection, and 3 (9.4%) required hospitalization for a median of 5 days (IQR 5-5) during the acute phase of infection. Thirty-five individuals who fully recovered from symptomatic acute SARS-CoV-2 infection were included as controls in the Recovered cohort. 57% participants with LC were diagnosed by RT-qPCR on nasopharyngeal swabs, while 43% were diagnosed by the presence of virus-specific IgM in plasma. In Recovered participants, 66% were diagnosed by PCR and 34% by serology. Most participants were women (97% in LC cohort, 71% in Recovered cohort) and the median age was 49 (IQR 45-52) and 50 years (IQR 38-52), respectively. The median time from COVID-19 diagnosis to sample collection was 26 months (IQR 23-27) for the LC cohort and 24 months (IQR 24-24) for the Recovered cohort. Key sociodemographic and clinical characteristics are in **Table 1**, with further details in **Supplementary Table 1** and **Supplementary Table 2**.

**Table 1:** Clinical and sociodemographic characteristics of all participants in this study.

	Long COVID (n=32)	Recovered (n=35)	P-value
Age, years; median (IQR)	49 (45-52)	50 (38-59)	0.9279
Female gender, n (%)	31 (97)	25 (71)	**0.0068**
Time from clinical onset to sample, months; median (IQR)	26 (23-27)	24 (24-24)	**0.0060**
Hospitalization, n (%)	3 (9.4)	0	0.1035
Time of hospitalization, days; median (IQR)	5 (5-5)	0	**-**
Long COVID symptoms (>3 months), n (%)			
Lack of concentration	27 (84)	0	**<0.0001**
Memory failure	27 (84)	0	**<0.0001**
Dizziness	15 (47)	0	**<0.0001**
Asthenia	30 (94)	0	**<0.0001**
General discomfort	23 (72)	0	**<0.0001**
Migraine	23 (72)	0	**<0.0001**
Low mood	20 (63)	0	**<0.0001**
Anxiety	14 (44)	6 (17)	**0.0312**
Muscle pain	25 (78)	0	**<0.0001**
Dyspnea	22 (69)	0	**<0.0001**
Joint pain	23 (72)	0	**<0.0001**
Back pain	24 (75)	0	**<0.0001**
Cervical pain	19 (59)	0	**<0.0001**
Chest pain	17 (53)	0	**<0.0001**
Chest tightness	16 (50)	0	**<0.0001**
Low-grade fever	5 (16)	0	**0.0209**
Cough	11 (34)	0	**0.0005**
Diarrhea	14 (44)	0	**<0.0001**
Palpitations	21 (66)	0	**<0.0001**
Tingling in extremities	20 (63)	0	**<0.0001**
Rashes	14 (44)	0	**<0.0001**
Comorbidities, Y/N/U; n (%)			
Diabetes mellitus	2 (6)/21 (66)/9 (28)	1 (3)/34 (97)/0	0.5565
Dyslipidemia	7 (22)/16 (50)/9 (28)	7 (20)/28 (80)/0	0.3622
Arterial hypertension	3 (9)/20 (63)/9 (28)	8 (23)/27 (77)/0	0.4986
Thyroid disorders	8 (19)/17 (53)/9 (28)	2 (6)/29 (83)/4 (11)	**0.0172**
Autoimmune disease	0/23 (72)/9 (28)	2 (6)/23 (66)/10 (29)	0.4902
Current treatments, n (%)			
Vitamins	7 (22)	1 (3)	**0.0261**
Asthma and allergic rhinitis	8 (25)	1 (3)	**0.0125**
Immunomodulators	2 (6)	3 (9)	1.0000
Analgesics/anti-inflammatories	8 (27)	3 (9)	0.1056
Antidepressants	9 (28)	7 (20)	0.5699
Anxiolytics	3 (9)	5 (14)	0.7072
Cardiovascular	7 (22)	8 (23)	1.0000
Vaccination against COVID-19, n (%)			
Participants who received at least 1 dose	28 (88)	32 (91)	0.1132
Participants who received 3 doses	10 (36)	28 (88)	**<0.0001**
Participants who received 2 doses	13 (46)	2 (6)	**0.0006**
Participants who received 1 dose	5 (18)	2 (6)	0.2349
Type of COVID-19 vaccine, n (%)			
Only Comirnaty® (Pfizer/BioNTech)	9 (32)	31 (97)	**<0.0001**
Only Spikevax® (Moderna)	0	0	1.0000
Only Vaxzevria® (Astrazeneca)	0	0	1.0000
Combination of vaccines	11 (39)	1 (3)	**0.0007**
Unknown vaccine	8 (29)	0 (0)	**0.0012**
Time from last vaccine dose to sampling, months; median (IQR)	8 (7-10)	4 (4-5)	**<0.0001**
SARS-CoV-2 breakthrough infections, n (%)	12 (38)	5 (14)	**0.0291**

IQR, interquartile range; N, no; U, unknown; Y, yes. Statistical significance between groups was calculated using Fisher´s exact test, chi-square test 2x2 and unpaired t test. Significant p values <0.05 are highlighted in bold font.

At the time of sampling, the most frequent symptoms reported by participants in the LC cohort were fatigue (94%), memory loss (84%), and difficulty concentrating (84%). Other common symptoms included back pain (75%), joint pain (72%), migraine (72%), general discomfort (72%), dyspnea (69%), palpitations (66%), tingling in the extremities (63%), low mood (63%), and neck pain (59%). LC participants also reported chest pain (53%), chest tightness (50%), dizziness (47%), skin rashes (44%), diarrhea (44%), and anxiety (44%). Less common symptoms include persistent cough (34%) and low-grade fever (16%). People with LC reported that these symptoms persisted for more than 3 months after COVID-19 diagnosis. In contrast, participants in the Recovered cohort did not report any of these symptoms for more than 3 months, except for anxiety, which was reported by 6 (17%) participants.

Comorbidities such as diabetes, dyslipidemia, and hypertension were recorded in both groups, with no significant differences observed. However, thyroid disorders were more common among LC participants compared to Recovered individuals (19% versus 6%; p=0.0172). Participants from both cohorts were receiving a variety of treatments, including immunomodulators, analgesics/anti-inflammatory drugs, antidepressants, anxiolytics, and/or cardiovascular treatments at sampling, with no significant differences between groups. A higher proportion of LC participants were on treatment for asthma and allergic rhinitis compared to the Recovered group (25% versus 3%; p=0.0096). Similarly, more LC participants were taking vitamin supplements than those in the Recovered group (22% versus 3%; p<0.0211).

Most participants in both LC and Recovered groups (88% and 91%, respectively) had received at least one dose of an authorized COVID-19 vaccine, with most having received two or three doses (94% and 82%, respectively). Among Recovered participants, the most common vaccine was Comirnaty® (Pfizer/BioNTech) (97%; p<0.0001), while LC participants received a combination of different vaccines (39%; p=0.0007). The LC cohort reported a higher incidence of breakthrough SARS-CoV-2 infections compared to the Recovered cohort (38% versus 14%; p=0.0291). Time from last vaccine dose to sampling was 8 (IQR 7-10) and 4 (IQR 4-5) months in LC and Recovered participants, respectively (p<0.0001). All breakthrough infections were mild, and no participant required hospitalization.

### 3.2 Detection of SARS-CoV-2 RNA in plasma and feces

The presence of RNA from SARS-CoV-2 was not detected in the plasma or feces of any participant in the study (data not shown).

### 3.3 Changes in the levels of antibodies against SARS-CoV-2 in people with LC

**Figure 1:**
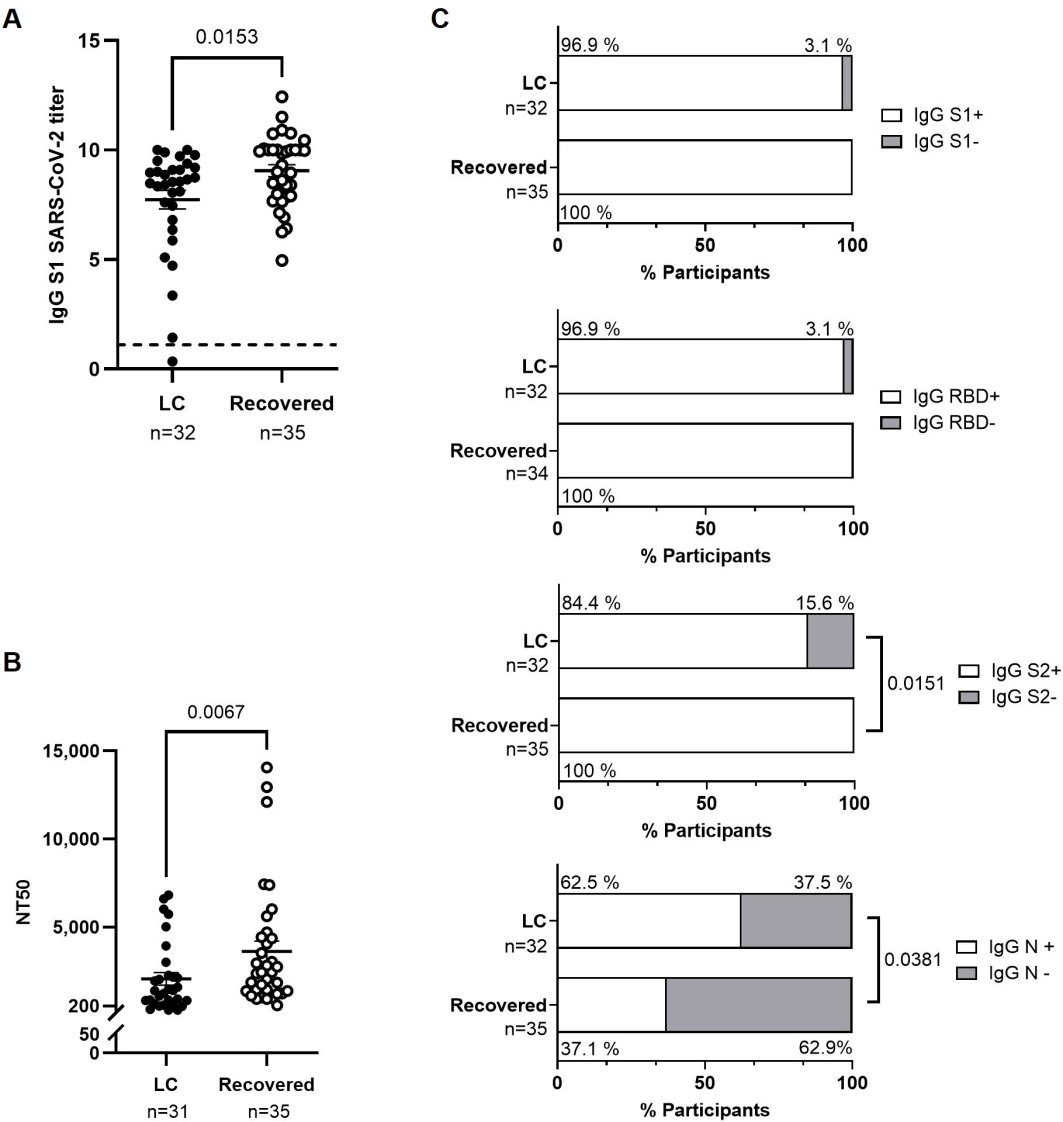
Characterization of the humoral response against SARS-CoV-2 in individuals with LC compared to Recovered. **(A)** Analysis by ELISA of the levels of IgG against SARS-CoV-2 spike protein subunit 1 (S1) in plasma from individuals with LC or Recovered. The horizontal line at 1.1 represents the positivity threshold. **(B)** Titers of neutralizing anti-S1 IgG in plasma calculated as NT50 from individuals with LC or Recovered. Each dot corresponds to one sample (LC, closed circles; Recovered, open circles) and vertical lines represent mean ± standard error of the mean (SEM). Statistical significance was obtained using non-parametric Mann-Whitney test. **(C)** Analysis by CLIA of the levels of IgG against S1, RBD, S2, and N proteins from SARS-CoV-2 in plasma of participants with LC or Recovered. Bars depict the percentage of participants considered producers (open bar) or non-producers (grey bar) of each IgG. Statistical significance was calculated using the Chi-square test.

We observed lower levels of IgG against the S1 protein in participants from the LC cohort compared to the Recovered cohort (-1.2-fold; p=0.0153) (**Figure 1A**). The neutralizing capacity of antibodies against SARS-CoV-2 was reduced 1.8-fold (p=0.0067) in LC cohort (**Figure 1B**). Analysis of the proportion of participants with detectable IgG against different viral proteins revealed that 15.6% of participants in the LC cohort had undetectable levels of IgG against the S2 protein, compared to the Recovered cohort (p=0.0151) (**Figure 1C**). In contrast, the number of participants in the LC cohort with detectable levels of IgG against the N protein was 1.7-fold higher than in the Recovered cohort (p=0.0381). No significant differences were found in the proportion of participants with detectable IgG against S1 and RBD.

### 3.4 Levels of plasma proteins related to intestinal barrier integrity and bacterial translocation

**Figure 2:**
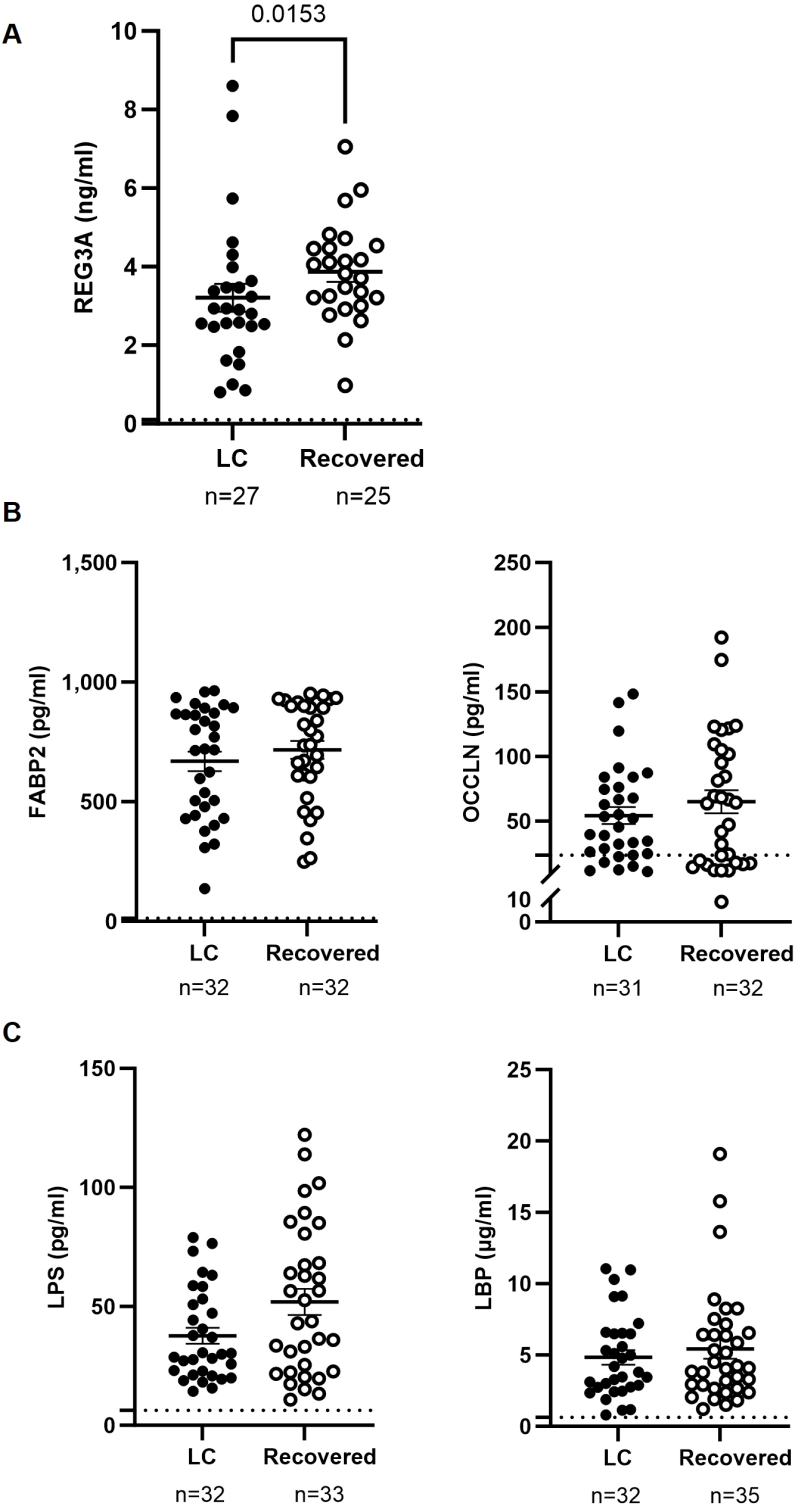
Analysis of factors related to intestinal injury and bacterial translocation in plasma of individuals with LC and Recovered. Plasma levels of the gut bactericidal protein REG3A **(A)**, markers of intestinal barrier integrity FABP2 and OCCLN **(B)**, and markers associated with bacterial translocation LPS and LBP **(C)**, were analyzed by ELISA. Each dot corresponds to one sample (LC, closed circles; Recovered, open circles) and vertical lines represent mean±SEM. Horizontal dashed lines mark the limit of detection. Statistical significance was calculated using non-parametric Mann-Whitney test.

Plasma levels of the bactericidal intestinal protein REG3A were 1.2-fold lower (p=0.0153) in the LC cohort compared to the Recovered cohort (**Figure 2A**). No significant differences were observed between groups in the levels of plasma proteins associated with intestinal injury, such as FABP2 and occludin (**Figure 2B**) or markers of bacterial translocation such as LPS and LBP (**Figure 2C**).

### 3.5 Plasma levels of cytokines and chemokines

**Figure 3:**
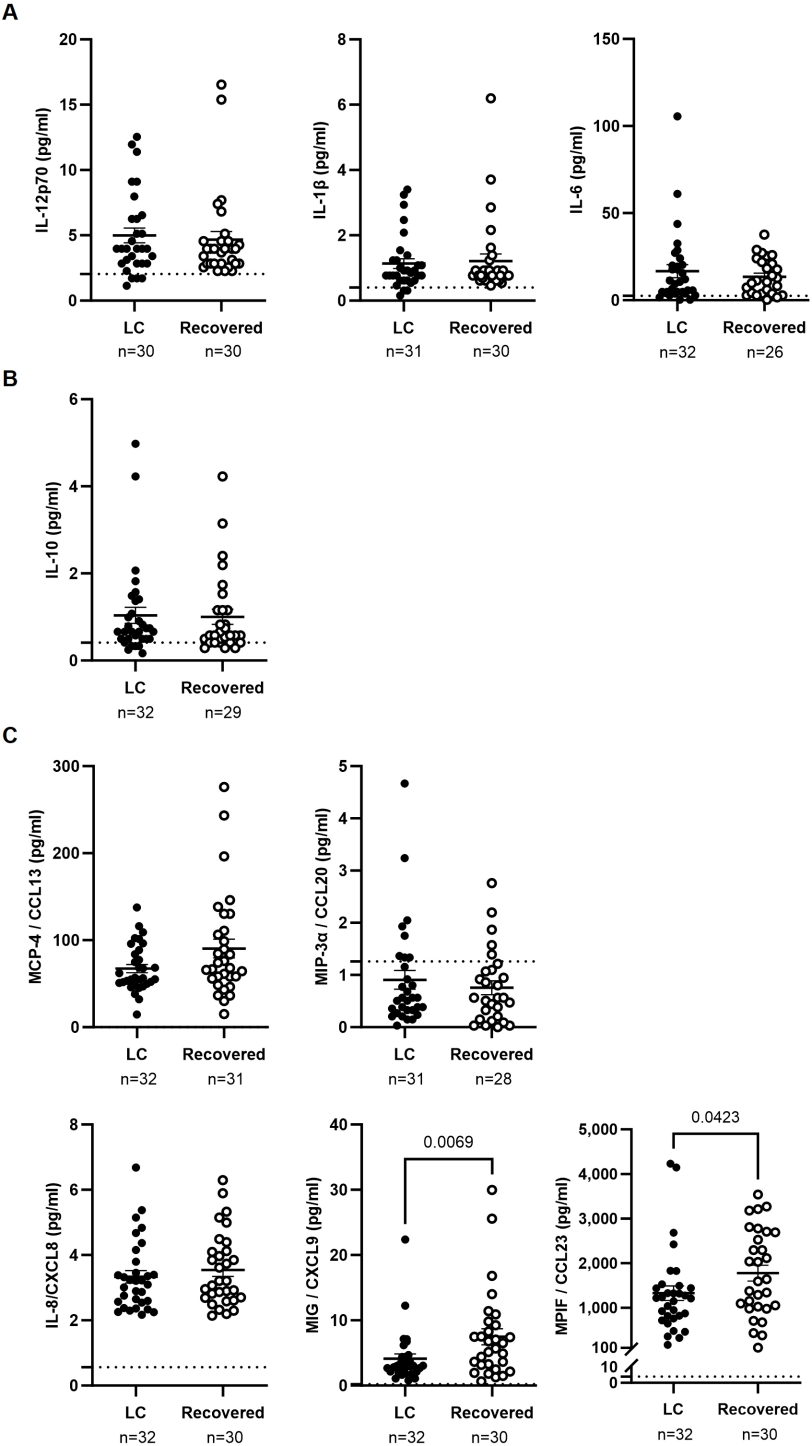
Analysis of cytokines in plasma of individuals with LC and Recovered. Plasma levels of proinflammatory cytokines IL-12p70, IL-1β, and IL-6 **(A)**, anti-inflammatory cytokine IL-10 **(B)**, and chemokines MCP-4/CCL1, MIP-3α/CCL20, IL-8/CXCL8, MIG/CXCL9, and MPIF/CCL23 **(C)** were analyzed by Luminex. Each dot corresponds to one sample (LC, closed circles; Recovered, open circles) and vertical lines represent mean±SEM. Horizontal dashed lines mark the limit of detection. Statistical significance was calculated using non-parametric Mann-Whitney test.

Similar levels of pro-inflammatory (IL-1β, IL-12, IL-6) (**Figure 3A**) and anti-inflammatory (IL-10) cytokines (**Figure 3B**) were observed in plasma of participants from both groups. However, participants in the LC cohort had 2.7- and 1.3-fold lower levels of the chemokines MIG/CXCL9 and MPIF/CCL23, respectively, compared to the Recovered cohort (p=0.0069 and p=0.0423), while levels of the chemokines MCP-4/CCL13, MIP-3α/CCL20, and IL-8/CXCL8 were similar between both groups (**Figure 3C**).

### 3.6 Serology and reactivation of herpesvirus

**Figure 4:**
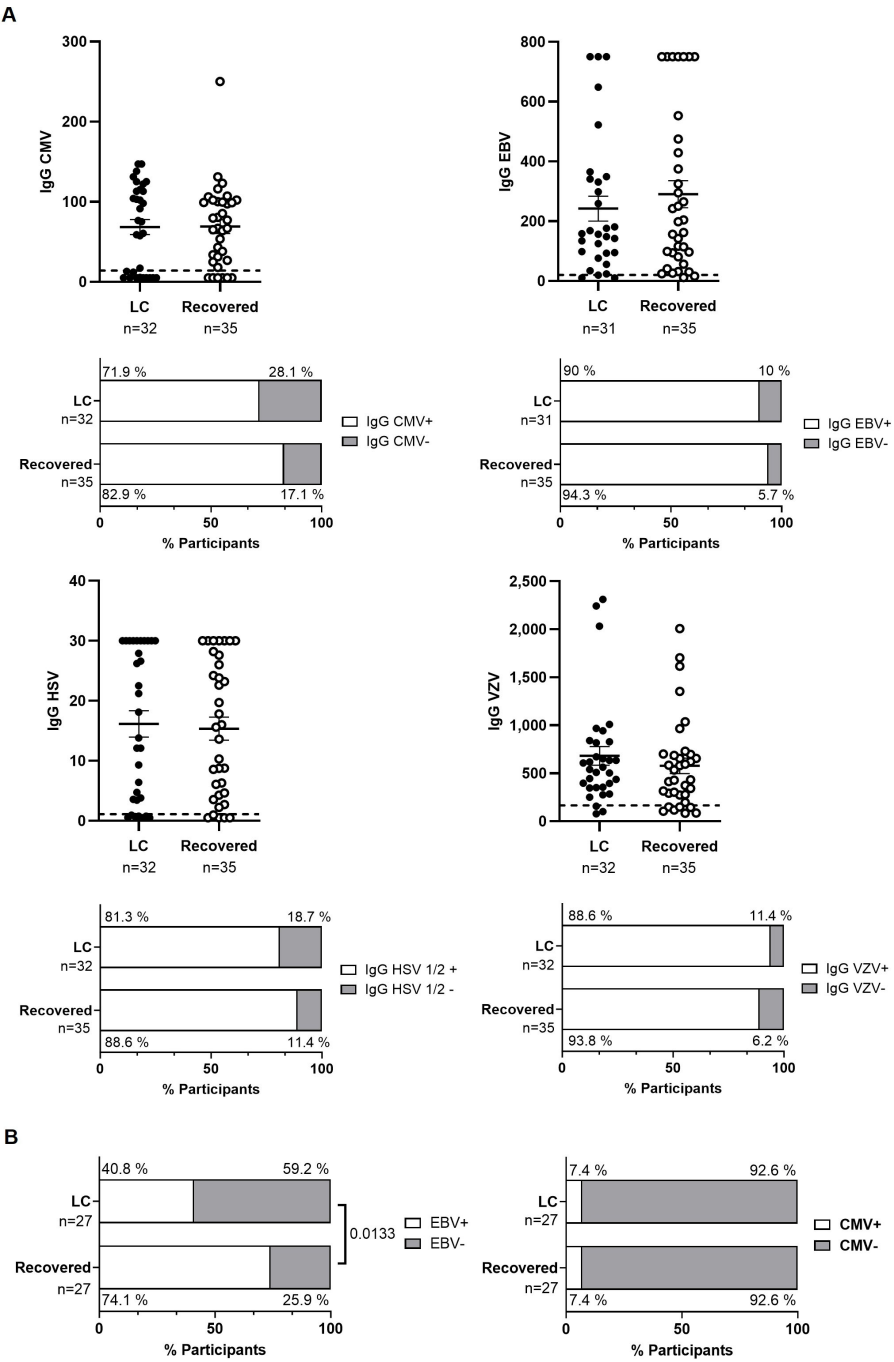
Analysis of herpesvirus reactivation in plasma from individuals with LC or Recovered. **(A)** Dot graphs show levels of IgG against CMV, EBV, HSV-1/2 and VZV analyzed by CLIA. Each dot represents data from one individual (LC, closed circles; Recovered, open circles) and vertical lines represent mean±SEM. Horizontal dashed line marks the limit of detection. Statistical significance was calculated using non-parametric Mann-Whitney test. Bar graphs show the count of individuals producers (open bar) or non-producers (grey bars) of antibodies against herpesvirus. Statistical significance was calculated using the Chi-square test. **(B)** Detection of provirus reactivation of EBV and CMV was analyzed by qPCR in plasma from participants with LC or Recovered. Graph bars depict the percentage of individuals in which these herpesviruses were reactivated (open bars) or not reactivated (grey bars). Statistical significance was calculated using the Chi-square test.

No significant differences were observed in plasma IgG titers against CMV, VZV, HSV-1/2, and EBV, nor in the total number of individuals who tested positive for each IgG between the two cohorts (**Figure 4A**). However, EBV DNA was detectable in the plasma of both the Recovered and LC cohorts (74.1% versus 40.8%, respectively; p=0.0133) (**Figure 4B**). Two individuals from each cohort (7.4%) had detectable CMV DNA in plasma. Reactivation of EBV or CMV did not result in any clinical consequences.

### 3.7 Evidence of endothelial dysfunction and altered coagulation in LC cohort

**Figure 5:**
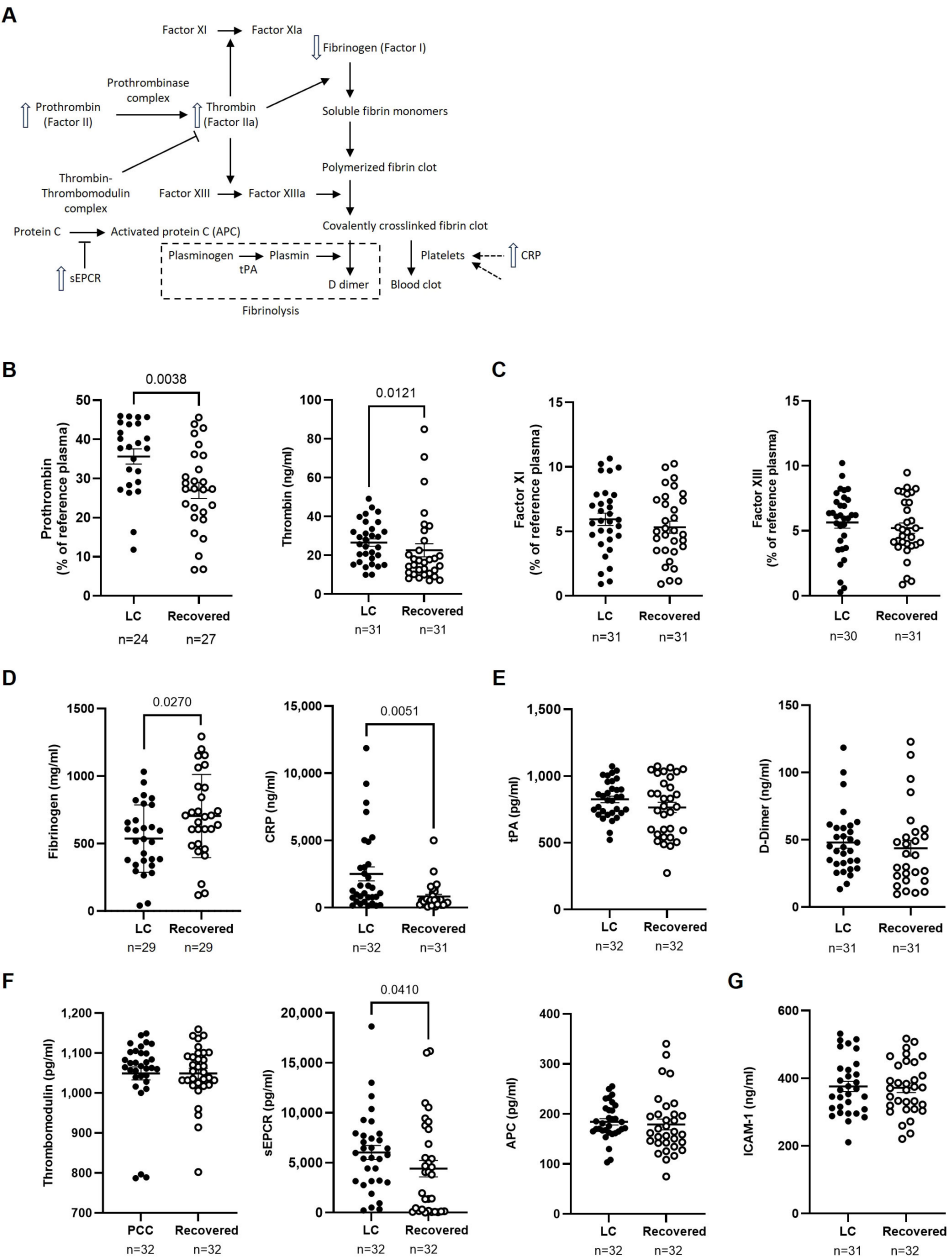
Analysis of markers of endothelial damage, thrombotic risk, and coagulation factors in the plasma of participants with LC or Recovered. **(A)** Schematic diagram of the interactions between coagulation proteins using thrombin as a central factor. Up and down arrows show those parameters that were increased or decreased, respectively, in plasma of individuals with LC compared to Recovered. **(B)** Analysis of plasma levels of prothrombin and thrombin as key proteins in the blood clotting process. **(C)** Analysis of plasma concentration of Factor XI, which is essential for the amplification phase of coagulation, and Factor XIII that is a fibrin-stabilizing factor. **(D)** Analysis of plasma concentration of acute-phase proteins fibrinogen and CRP whose levels increase during inflammation and acute responses. **(E)** Analysis of plasma levels of fibrinolysis markers tPA (tissue plasminogen activator) and D-dimer, which are associated with the breakdown of clots. **(F)** Analysis of plasma levels of anticoagulant regulators thrombomodulin, sEPCR (soluble Endothelial Protein C Receptor), and APC (Activated Protein C), which are involved in the regulation of anticoagulation and maintaining hemostatic balance. **(G)** Analysis of plasma concentration of endothelial activator ICAM-1 that can influence coagulation processes in the context of inflammation and thrombosis. Each dot represents data from one individual (LC, closed circles; Recovered, open circles) and vertical lines represent mean±SEM. Statistical significance was obtained using non-parametric Mann-Whitney test or unpaired t-test, as appropriate.

The expression of several proteins related to endothelial dysfunction and coagulation was analyzed in plasma using thrombin as a central factor (**Figure 5A**). Individuals from the LC cohort showed significantly higher levels of prothrombin (1.3-fold; p=0.0038) and thrombin (1.2-fold; p=0.0121) compared to the Recovered cohort (**Figure 5B**). Since thrombin mediates activation of Factors XI and XIII (30), we also assessed the levels of these factors, but no significant changes were observed between the cohorts (**Figure 5C**). Thrombin cleaves fibrinogen to form fibrin monomers, which then polymerize into a fibrin clot (31). As a result, fibrinogen levels were 1.3-fold lower (p=0.0270), while CRP levels were 3.1-fold higher (p=0.0051) in LC participants, alterations that have been previously linked to a higher risk of venous thromboembolism (32) (**Figure 5D**). No significant differences were observed in tPA levels, which mediate plasminogen conversion during fibrinolysis (33), or in D-dimer levels, a degradation product of cross-linked fibrin (34) (**Figure 5E**).

Since the thrombin-thrombomodulin complex activates PC to downregulate coagulation and inflammation (35), we also analyzed plasma levels of thrombomodulin and APC and found no differences between the two groups. However, sEPCR, which inhibits APC activity and has been reported as a marker associated with higher thrombotic risk (36), was 1.4-fold higher (p=0.0410) in the LC cohort compared to the Recovered cohort (**Figure 5F**).

Lastly, there were no differences in the plasma levels of ICAM-1 (**Figure 5G**), an endothelial injury marker (37), between the two groups.

### 3.8 Association of qualitative and quantitative variables with the persistence of COVID-19

The association of the qualitative variables in the development of LC was analyzed using binary logistic regression analysis (OR) (**Table 2A**). This analysis revealed that female gender (OR 12.400; 95% CI 1.485 to 103.520; p=0.020), receiving doses of a combination of different vaccines rather than the same type (OR 5.775; 95% CI 1.991 to 16.748; p=0.001), high levels of antibodies against the SARS-CoV-2 N protein (OR 2.821; 95% CI 1.047 to 7.599; p=0.040), and the development of asthma and/or allergic rhinitis (OR 10.909; 95% CI 1.270 to 93.692; p=0.029) were positively correlated with the occurrence of LC. Additionally, LC participants were more likely to take vitamin supplements compared to the Recovered cohort (OR 9.130; 95% CI 1.048 to 79.532; p=0.045).

Table 2Association between qualitative (A) and quantitative variables (B) with the development of LC was assessed using simple linear regression analysis and subsequent binary logistic regression analysis to calculate odds ratio (OR) for quantitative variables and using logistic regression analysis for qualitative variables.

**Table:** 

A) Qualitative variables			
Variable	OR	P-value	Binary logistic
			95% CI
Female gender	12.4	**0.02**	1.485 to 103.520
Diabetes mellitus	3.143	0.362	0.267 to 36.860
Dyslipidemia	1.688	0.399	0.500 to 5.696
Arterial hypertension	0.7	0.641	0.156 to 3.137
Thyroid disorders	4.028	0.117	0.705 to 22.996
Vitamins	9.13	**0.045**	1.048 to 79.532
Analgesics/anti-inflammatories	3.394	0.096	0.804 to 14.319
Cardiovascular treatment	0.875	0.823	0.272 to 2.812
Asthma/allergic rhinitis treatment	10.909	**0.029**	1.270 to 93.692
Anti-depressants	1.469	0.511	0.466 to 4.632
Anxiolytics	0.578	0.482	0.125 to 2.666
Immunomodulators	0.322	0.339	0.032 to 3.281
Vaccine combination	5.775	**0.001**	1.991 to 16.748
Breakthrough infections	3	0.072	0.906 to 9.936
Anxiety	3	0.052	0.992 to 9.067
Positive for SARS-CoV-2 anti-N IgG	2.821	**0.04**	1.047 to 7.599
EBV reactivation	0.241	**0.015**	0.076 to 0.762
CMV reactivation	1	1	0.130 to 7.667

CMV, Cytomegalovirus; EBV, Epstein-Barr virus. Significant p values <0.05 are highlighted in bold font.

**Table:** 

B) Quantitative variables						
Variable	β	P-value	Simple linear 95% CI	OR	P-value	Binary logistic 95% CI
Age	-0.001	0.930	-0.013 to 0.012			
Number of vaccine doses	-0.283	**<0.001**	-0.412 to -0.153	0.194	**0.001**	0.074 to 0.511
Time from clinical onset to sample	0.019	0.390	-0.025 to 0.064			
SARS-CoV-2 anti-S1 IgG titer	-0.075	**0.010**	-0.131 to -0.019	0.691	**0.019**	0.508 to 0.941
NT50	-0.005	**0.030**	-0.009 to -0.0004	0.977	**0.046**	0.954 to 1.000
IgG CMV titer	<0.001	0.963	-0.002 to 0.002			
IgG EBV titer	<0.001	0.444	-0.001 to 0.0003			
IgG HSV titer	0.001	0.787	-0.009 to 0.012			
IgG VZV titer	<0.001	0.410	-0.0001 to 0.0003			
IL-1β	-0.019	0.769	-0.149 to 0.111			
IL-6	0.003	0.486	-0.005 to 0.011			
IL-8/CXCL8	-0.044	0.453	-0.162 to 0.073			
IL-10	0.009	0.898	-0.124 to 0.141			
IL-12p70	0.008	0.703	-0.033 to 0.049			
MCP-4/CCL13	-0.003	0.054	-0.005 to 0.00004			
MIP3α/CCL20	0.051	0.509	-0.103 to 0.205			
MIG/CXCL9	-0.026	**0.020**	-0.048 to -0.004	0.869	**0.042**	0.759 to 0.995
MPIF/CCL23	-0.139	**0.035**	-0.267 to -0.010	0.552	**0.042**	0.311 to 0.979
REG3A	-0.065	0.142	-0.152 to 0.022			
FABP2	<0.001	0.386	-0.001 to 0.0003			
OCCLN	-0.001	0.337	-0.004 to 0.001			
LPS	-0.005	**0.032**	-0.010 to -0.001	0.979	**0.038**	0.959 to 0.999
LBP	-0.014	0.439	-0.050 to 0.022			
Prothrombin	0.018	**0.004**	0.006 to 0.030	1.090	**0.008**	1.023 to 1.161
Thrombin	0.004	0.124	-0.001 to 0.010			
Factor XI	0.022	0.364	-0.027 to 0.072			
Factor XIII	0.020	0.470	-0.035 to 0.075			
Fibrinogen	-0.004	0.080	-0.008 to 0.001			
CRP	0.078	**0.004**	0.027 to 0.129	1.760	**0.018**	1.100 to 2.814
tPA	<0.001	0.190	-0.0002 to 0.001			
D-Dimer	0.001	0.555	-0.004 to 0.006			
Thrombomodulin	<0.001	0.990	-0.002 to 0.002			
sEPCR	0.022	0.139	-0.007 to 0.050			
APC	0.001	0.678	-0.002 to 0.003			
ICAM-1	<0.001	0.845	-0.001 to 0.002			

CMV, Cytomegalovirus; EBV, Epstein-Barr virus; HSV, Herpes Simplex virus; NT50, 50% Neutralization Titer; VZV, Varicella Zoster virus.

The association of the quantitative variables in the development of LC was assessed through linear regression followed by binary logistic regression analysis (**Table 2B**). Linear regression revealed a trend toward an association between the number of COVID-19 vaccine doses, the neutralizing capacity of IgG against SARS-CoV-2, the titers of IgG against SARS-CoV-2 S1 protein, and plasma levels of CXCL9, CCL23, prothrombin, and CRP with the development and/or persistence of LC, compared to healthy donors. This trend was confirmed by binary logistic regression, indicating that low number of vaccine doses (OR 0.194; 95% CI 0.074 to 0.511; p=0.001), IgG levels against SARS-CoV-2 S1 protein (OR 0.691; 95% CI 0.508 to 0.941; p=0.019), neutralizing capacity (NT50) of IgG against SARS-CoV-2 (OR 0.977; 95% CI 0.954 to 1.000; p=0.046), MIG/CXCL9 levels (OR 0.869; 95% CI 0.759 to 0.995; p=0.042), MPIF/CCL23 levels (OR 0.552; 95% CI 0.311 to 0.979; p=0.042) were correlated with the occurrence of LC. Conversely, high prothrombin levels (OR 1.090; 95% CI 1.023 to 1.161; p=0.008) and CRP levels (OR 1.760; 95% CI 1.100 to 2.814; p=0.018) were correlated with the occurrence of LC.

### 3.9 Application of random forest for the evaluation of the importance of the assayed variables for LC

**Figure 6:**
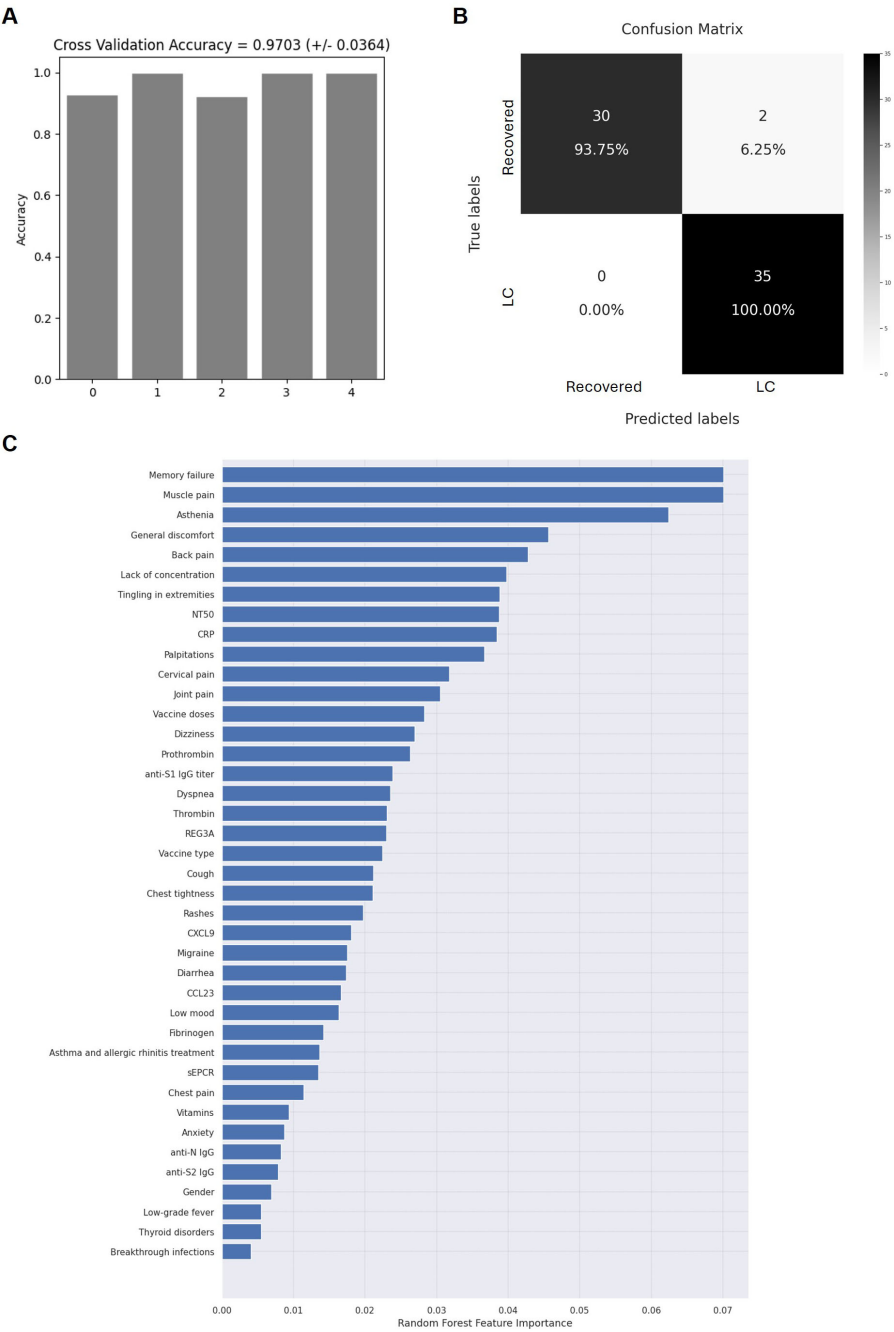
Application of random forest algorithm and Gini VIM method to evaluate the accuracy and importance of the selected biomarkers. Accuracy for the 5 iterations of the outer loop of the nested K-fold cross validation **(A)** and confusion matrix confronting the conditions calculated by the algorithm and the true chronicity-related conditions of individuals with LC or Recovered **(B, C**) Relative importance of the selected parameters for the categorization of individuals with LC or Recovered, according to Gini VIM method.

An accuracy of 97.03% ± 3.64% was achieved across the 5 iterations of the outer loop in the nested K-fold cross-validation for each competing algorithm (**Figure 6A**). As a result, all 35 participants (100%) in the LC cohort were correctly classified, while 30 of the 32 participants (93.75%) in the Recovered group were accurately assigned to their respective group (**Figure 6B**). The Gini VIM method identified several clinical variables as crucial for classification into the LC group, including memory failure and lack of concentration, muscle and back pain, paresthesia in extremities, asthenia, and malaise (**Figure 6C**). Key quantitative variables contributing to LC group classification included the neutralizing capacity of IgGs against SARS-CoV-2, the levels of CRP, thrombin, prothrombin, and REG3A, as well as the number of vaccine doses and having received a combination of vaccine types rather than a single vaccine type. Conversely, variables with lower importance for LC group assignment included gender, low-grade fever, thyroid disorders, and having had breakthrough infections.

## 4 Discussion

Long COVID (LC) has emerged as a major sequela of the COVID-19 pandemic, presenting challenges for diagnosis and treatment. Despite extensive research, the precise causes remain unclear, and among the more than 200 reported symptoms, debate persists regarding which are most critical for diagnosis and prognosis. Identifying key biomarkers is essential to understanding LC mechanisms and guiding effective treatments.

Key contributors to LC include host-related and external factors such as SARS-CoV-2 persistence, herpesvirus reactivation, and dysbiosis due to endothelial damage. Demographic factors like middle age and female gender have been consistently linked to LC susceptibility (3, 5, 38). In our cohort, 97% of LC participants were women (median age: 49 years). Most (90.6%) did not require hospitalization during acute infection, confirming that LC is not limited to severe cases. While comorbidities like diabetes, dyslipidemia, and hypertension have been associated with LC (5), we found no significant differences in their prevalence between LC and Recovered individuals, except for thyroid disorders (19% vs. 6%; p=0.0172). This finding aligns with previous reports, but the lack of baseline data raises questions about whether thyroid dysfunction results from an autoimmune response triggered by SARS-CoV-2 infection, vaccination, or treatments (39, 40). While both thyroid disorders and LC are more common among women, studies that controlled for age and gender still reported a higher prevalence of thyroid dysfunction in LC cohorts (39). Finally, key symptoms included asthenia, memory failure, concentration issues, muscle and joint pain, migraines, dyspnea, and palpitations; and 44% of LC participants reported low mood and anxiety, compared to 17% in the Recovered group (4).

An effective immune response is crucial for controlling SARS-CoV-2 primary infection and preventing reinfections. Impairments in both humoral and cellular immunity have been documented in COVID-19 (25, 41, 42) and LC (43–45). Our LC cohort exhibited lower SARS-CoV-2-specific IgG levels and reduced neutralization capacity. One participant had undetectable IgG levels despite a positive qPCR diagnosis, and another had IgG just above the threshold despite receiving two Comirnaty® doses. This impaired humoral response may increase susceptibility to reinfections, potentially exacerbating or perpetuating the condition. LC individuals from our cohort experienced a higher frequency of breakthrough infections compared to Recovered participants, which may contribute to the persistence of symptoms characteristic of LC (46, 47). This finding remark that an adequate vaccination schedule is essential to develop a protective humoral immunity, although not all antibodies are equally produced in vaccinated individuals. Elevated anti-N IgG levels are not directly related to vaccination and they suggest either recent reinfections or persistent viral proteins in LC individuals (48). Although these results remain controversial (49), high levels of anti-N IgG have been previously associated with LC and related neurological symptoms (50), as well as with the persistence of viral proteins in the host (51). Vaccination predominantly induces IgG against the S1 subunit and RBD, with lower induction of anti-S2 antibodies, likely because the S2 subunit is not fully exposed until the protein interacts with the ACE2 receptor (52). Interestingly, individuals vaccinated with booster doses exhibit increased levels of anti-S2 IgG compared to those with natural infection alone (53). In our LC cohort, not all individuals tested positive for anti-S2 IgG, and some presented low levels of anti-S1 IgG and reduced neutralization capacity. This implied that vaccination may have induced a less effective humoral response in LC individuals, increasing their susceptibility to reinfections. Although reinfections should also reinforce immunity against SARS-CoV-2 better than vaccination, the reinfections themselves pose additional risks, often contributing to further sequelae in multiple organ systems beyond those observed during the initial infection (54). Remarkably, 39% of LC participants had received mixed vaccine types, compared to only 3% of Recovered individuals, identifying vaccine heterogeneity as a potential LC risk factor. Together, these findings emphasize the critical role of an effective and consistent vaccination strategy in reducing the risk of LC and associated complications.

LC-associated immune dysregulation may stem from suboptimal vaccination, unresolved infection, or persistent immune activation due to viral reservoirs (9). While prolonged viral shedding in feces has been documented (11), we did not detect viral RNA in plasma or stool, likely due to the long interval (26 months) between infection and sample collection. Although viral proteins may persist in tissues or extracellular vesicles (55–57), intestinal dysbiosis may also contribute to sustained immune activation (58, 59). In our cohort, 44% of LC individuals reported persistent diarrhea. Though no direct signs of increased gut permeability were detected, REG3A levels, critical for epithelial regeneration, were significantly reduced in LC individuals (60, 61). REG3A deficiency promotes inflammation and alters microbiota (62), potentially exacerbating LC symptoms and contributing to persistent inflammation (13, 58). This correlates with the lower capacity of CD4+ Th22 cells to release IL-13 and IL-22 in people with LC (44). Th22 cells play a re-epithelializing role through IL-22 production (63) and both IL-13 and IL-22 are protective factors during acute and persistent COVID-19, promoting tissue protection and regeneration (64). Additionally, LC participants exhibited lower levels of MIG/CXCL9, MPIF/CCL23, and MPIF/CCL2, suggesting impaired immune recruitment and delayed inflammation resolution (65, 66). These deficits align with a detrimental Th1 antiviral response reported in LC (44). Psychological factors, including anxiety and depression (reported by 50% of LC cohort), could also contribute to gastrointestinal symptoms (67). Overall, these findings suggest that, while we did not observe direct signs of bacterial translocation or dysbiosis, an impaired mucosal immune axis may underlie gastrointestinal dysregulation in LC.

Persistent immune activation in LC could lead to herpesvirus reactivation (9), yet we found no significant EBV, CMV, VZV, or HSV-1/2 reactivation in LC individuals, possibly due to their heightened cytotoxic response (45). Thus, LC appears more closely associated with a sustained, uncontrolled inflammatory response rather than immunodeficiency. When the levels of pro- and anti-inflammatory markers were analyzed, CRP, a marker elevated in response to infections, tissue damage, and various inflammatory conditions (68), was increased in plasma of LC participants. CRP can remain elevated for months after acute COVID-19 (16, 69) and it has been associated with cardiovascular events due to its interaction with endothelial cells and the coagulation system (70). COVID-19 has been linked to cardiovascular disorders like myocardial injury, arrhythmia, and venous thromboembolism, due to the virus's affinity for endothelial cells (71). Several reports have identified markers of endothelial damage, microclots, complement dysregulation, thromboinflammation, and hypercoagulability in LC individuals (17, 72). In our study, prothrombin and thrombin levels were increased in plasma of LC individuals (73). Both thrombin and CRP are associated with endothelial activation and dysfunction during acute COVID-19 (74). In LC individuals, elevated thrombin levels coincided with decreased fibrinogen, a pattern that has been previously associated with an increased thrombotic risk in acute COVID-19 (17, 75). Our analysis revealed no compensatory mechanisms for this alteration, as fibrinolytic activity and the levels of the marker of fibrin degradation D-dimer remained unchanged. Additionally, LC participants showed higher plasma levels of sEPCR, which reduces the anticoagulant and anti-inflammatory effects of APC by competing with membrane-bound EPCR (36). Elevated sEPCR levels have been reported in acute severe COVID-19 and other chronic inflammatory conditions such as systemic lupus erythematosus (76), but this is the first report of increased sEPCR in plasma of LC individuals. Higher sEPCR levels may result from vascular injury or regulated proteolytic release, possibly through thrombin-induced endothelial stimulation and subsequent metalloproteinase activity (77). These findings support the persistent endothelial and coagulation dysfunctions in LC.

Although no pre-pandemic uninfected control group was available to establish basal levels of inflammatory markers, most of these markers typically normalize within 8–10 months after acute infection (78–81). Therefore, our Recovered group can be considered an adequate control, as a median of 24 months had elapsed since the acute infection. In addition, reinfections reported in some participants occurred more than 6 months before sampling, making it unlikely that they influenced the immunological and endothelial alterations detected in the LC cohort.

Among the potential limitations of this study, we must consider the differences in gender distribution, since LC predominantly affects women (53). However, as this was an observational cohort rather than a case-control study, we chose to preserve the natural female predominance in LC and to recruit a control group representative of the broader post-COVID population, rather than artificially adjusting its sex composition. This approach avoids selection bias and provides a realistic comparator group, while still allowing meaningful identification of LC-associated biological features. Other limitation is the fact that some symptoms were self-reported, which could be especially important for the cognitive manifestations that were not recorded through standardized neurocognitive tests, which may limit their objective quantification. However, the use of self-reported symptoms is broadly accepted in LC research, given that many manifestations of the syndrome (e.g., fatigue, brain fog, malaise, or pain) are inherently subjective and often not fully captured by standardized tests available in routine practice. In addition, some clinical variables used in the Random Forest analysis could be considered part of the original criteria for classifying participants as LC, which may introduce circularity and limit the interpretation of the model as an independent predictive tool. Nevertheless, including these clinical variables is important, as it allows the model to capture the central features defining LC and to identify biological markers that cluster with them, providing an integrated view of the syndrome's multidimensional profile.

In conclusion, the novelty of this study lies in its long-term, multidimensional analysis of LC-related parameters. By examining immune dysregulation and persistent endothelial damage, we identified a combined mechanism associated with coagulation abnormalities that may contribute to a pro-thrombotic profile in LC. These findings suggest that, despite impaired humoral and mucosal immunity, LC could be more strongly linked to a sustained, uncontrolled inflammatory response rather than to immunodeficiency, and appears to illuminate underlying pathogenic processes that could go beyond traditional comorbidities and challenge earlier models based on isolated pathways. Uniquely, our work also uncovers an association between vaccine heterogeneity and LC, suggesting that inconsistent vaccination strategies may compromise immune protection and contribute to the syndrome, thus emphasizing the need for uniform vaccination policies. Furthermore, the study's strength is underscored by using an exploratory Random Forest model that achieved 100% accuracy in distinguishing LC from recovered individuals, emphasized the association between key clinical features and related biological markers. This multidimensional approach not only advances our understanding of LC's complex pathogenesis but also establishes a solid foundation for enhanced diagnostic tools, ultimately leading to better prevention and clinical management strategies for affected individuals.

## Data Availability

The original contributions presented in the study are included in the article/**Supplementary Material**. Further inquiries can be directed to the corresponding authors.
