# Supplementary material for: Immune dysregulation and endothelial dysfunction associate with a pro-thrombotic profile in Long COVID
Source: Front Immunol. 2025 Oct 16;16:1613195. doi: 10.3389/fimmu.2025.1613195 (PMC12571756; doi:10.3389/fimmu.2025.1613195)
Supplement: Supplementary file 1 [file Table1.docx]

**Supplemental Table 1.** Sociodemographical and clinical characteristics of participants with LC.

| **ID** | **Age (years)** | **Gender (M/F)** | **Time from clinical onset to sample (months)** | **Hospitalization due to acute COVID-19**  **(Y/N; days)** | **Comorbidities (Y/N)** | | | | | **Current treatments**  **(Y/N; which)** | **COVID-19 vaccines**  **(Y/N; which)** | **Doses of COVID-19 vaccines (no.)** | **Breakthrough infection**  **(Y/N; no.)** |
| --- | --- | --- | --- | --- | --- | --- | --- | --- | --- | --- | --- | --- | --- |
|  |  |  |  |  | **DM** | **DL** | **HTA** | **TD** | **AID** |  |  |  |  |
| **1** | 52 | M | 17 | N | N | N | N | N | N | N | Y; Comirnaty®/Comirnaty®/Spikevax® | 3 | N |
| **2** | 51 | F | 26 | N | N | Y | N | N | N | Y; VIT/AA | Y; Comirnaty®/Comirnaty® | 2 | N |
| **3** | 45 | F | 27 | N | N | N | N | N | N | Y; AA | N | 0 | N |
| **4** | 47 | F | 10 | N | N | N | N | N | N | Y; VIT | Y; Comirnaty®/Comirnaty® | 2 | Y; 2 |
| **5** | 53 | F | 27 | N | N | Y | Y | N | N | Y; VIT/AA | Y; U | 1 | Y, 1 |
| **6** | 39 | F | 17 | N | N | N | N | Y | N | Y; AA/AN/AD/CAR | Y; Comirnaty®/Comirnaty® | 2 | Y; 1 |
| **7** | 45 | F | 28 | N | N | N | N | N | N | Y; AN | Y; Comirnaty®/Comirnaty® | 2 | N |
| **8** | 41 | F | 21 | N | N | Y | N | Y | N | Y; AD/AL/CAR | Y; Spikevax®/Spikevax®/Comirnaty® | 3 | N |
| **9** | 55 | F | 19 | N | U | U | U | U | U | Y; CAR | Y; U | 2 | Y; 1 |
| **10** | 45 | F | 28 | N | N | N | N | N | N | Y; AA/AN | Y; Comirnaty® | 1 | Y; 1 |
| **11** | 52 | F | 26 | N | U | U | U | U | U | Y; VIT | Y; U | 2 | N |
| **12** | 50 | F | 24 | N | U | U | U | U | U | Y; AA | Y; U | 1 | N |
| **13** | 34 | F | 27 | N | N | N | N | N | N | Y; IM | Y; Comirnaty®/Spikevax® | 2 | N |
| **14** | 46 | F | 27 | N | N | N | N | N | N | Y; AD | Y; Spikevax®/Spikevax®/Comirnaty® | 3 | N |
| **15** | 46 | F | 27 | N | N | N | N | N | N | N | Y; Comirnaty® | 1 | Y; 1 |
| **16** | 56 | F | 24 | N | N | N | N | Y | N | Y; AN | Y; Comirnaty®/Comirnaty® | 2 | N |
| **17** | 41 | F | 26 | N | Y | Y | N | N | N | Y; AN/AD | Y; Vaxzevria®/Comirnaty®/Spikevax® | 3 | Y; 1 |
| **18** | 42 | F | 27 | N | N | N | N | N | N | Y; AN | Y; Comirnaty®/Spikevax® | 2 | Y; 1 |
| **19** | 60 | F | 27 | N | N | N | N | Y | N | Y; VIT/AD/CAR | Y; Comirnaty®/Comirnaty®/Spikevax® | 3 | Y; 1 |
| **20** | 61 | F | 24 | N | N | Y | Y | Y | N | Y; AA/AN/AD | Y; Comirnaty®/Spikevax® | 2 | N |
| **21** | 48 | F | U | N | U | U | U | U | U | N | Y; U | 2 | N |
| **22** | 53 | F | 25 | N | N | N | N | N | N | Y; VIT/CAR | Y; Comirnaty®/Comirnaty® | 2 | N |
| **23** | 52 | F | 27 | N | N | N | Y | N | N | Y; AD/AL/CAR | Y; Comirnaty®/Comirnaty®/Spikevax® | 3 | N |
| **24** | 46 | F | 28 | N | U | U | U | Y | U | N | Y; U | 1 | Y; 1 |
| **25** | 50 | F | 27 | N | N | N | N | N | N | N | N | 0 | N |
| **26** | 45 | F | 24 | N | U | U | U | U | U | N | Y; U | 3 | N |
| **27** | 49 | F | 21 | Y, 5 | N | Y | N | N | N | Y; AD | Y; Comirnaty®/Spikevax®/Comirnaty® | 3 | N |
| **28** | 53 | F | 28 | Y, 5 | N | N | N | N | N | N | Y; Comirnaty®/Spikevax® | 2 | Y; 1 |
| **29** | 49 | F | 27 | Y, 5 | U | U | U | U | U | Y; AN/AD/AL | N | 0 | N |
| **30** | 46 | F | U | N | U | U | U | U | U | Y; VIT/AA/CAR | Y; U | 3 | Y; 1 |
| **31** | U | F | U | U | U | U | U | U | U | U | U | U | N |
| **32** | U | F | U | U | Y | Y | N | N | N | U | Y; Comirnaty®/Comirnaty®/Comirnaty® | 3 | N |

AA: asthma and allergic rhinitis; AD: anti-depressants; AID: autoimmune disease; AL: anxiolytics; AN: analgesics/anti-inflammatories; CAR: cardiovascular; DL: dyslipidemia; DM: diabetes mellitus; F: female; HTA: hypertension; IM: immunomodulators; M: male; N: no; TD: thyroid disorders; U: unknown; VIT: vitamins; Y: yes.

**Supplemental Table 1 (continuation)**

| **ID** | **LC signs and symptoms (Y/N)** | | | | | | | | | | | | | | | | | | | | |
| --- | --- | --- | --- | --- | --- | --- | --- | --- | --- | --- | --- | --- | --- | --- | --- | --- | --- | --- | --- | --- | --- |
|  | **LOC** | **MF** | **DIZ** | **AST** | **GD** | **MI** | **LM** | **ANX** | **MP** | **DYS** | **JP** | **BP** | **CP** | **CHP** | **CHT** | **LGF** | **CO** | **DIA** | **PAL** | **TIN** | **RAS** |
| **1** | Y | Y | Y | Y | N | N | Y | Y | Y | N | N | Y | Y | N | N | N | N | N | N | Y | N |
| **2** | N | Y | N | Y | N | N | Y | N | Y | Y | Y | N | N | Y | Y | Y | N | Y | Y | N | Y |
| **3** | Y | Y | Y | Y | Y | Y | N | N | N | N | Y | N | N | N | N | N | N | N | N | Y | N |
| **4** | Y | Y | N | Y | Y | Y | N | N | Y | Y | Y | Y | Y | Y | Y | N | Y | Y | Y | N | N |
| **5** | Y | Y | Y | Y | Y | Y | Y | N | Y | N | Y | Y | Y | N | N | Y | N | N | Y | Y | N |
| **6** | Y | Y | Y | Y | Y | Y | Y | N | Y | Y | N | N | N | Y | N | N | N | N | Y | N | Y |
| **7** | N | N | N | Y | Y | Y | N | N | Y | Y | Y | Y | N | Y | Y | N | N | Y | Y | N | Y |
| **8** | Y | Y | Y | Y | Y | Y | N | Y | Y | N | N | Y | N | Y | N | N | N | N | Y | Y | N |
| **9** | Y | Y | Y | Y | Y | Y | Y | Y | N | Y | N | Y | Y | Y | Y | N | N | N | Y | Y | N |
| **10** | N | Y | N | Y | Y | N | N | N | Y | Y | Y | Y | Y | N | N | Y | N | N | Y | Y | Y |
| **11** | Y | N | N | Y | Y | Y | Y | Y | Y | Y | Y | Y | Y | Y | Y | N | Y | Y | Y | Y | Y |
| **12** | Y | Y | N | Y | Y | Y | Y | N | Y | N | N | N | N | N | N | Y | Y | N | N | Y | N |
| **13** | Y | Y | N | Y | N | N | Y | N | Y | Y | Y | Y | N | N | Y | N | N | N | Y | N | Y |
| **14** | Y | Y | N | Y | Y | Y | Y | Y | Y | Y | Y | Y | Y | Y | Y | N | N | Y | Y | Y | Y |
| **15** | Y | Y | N | Y | Y | N | Y | N | Y | Y | Y | Y | Y | Y | Y | N | N | Y | Y | Y | Y |
| **16** | Y | Y | Y | Y | N | Y | Y | Y | Y | Y | Y | Y | Y | N | N | N | N | Y | N | Y | Y |
| **17** | Y | N | N | Y | Y | Y | N | N | Y | Y | Y | Y | N | Y | N | N | N | N | N | Y | N |
| **18** | Y | Y | N | Y | N | Y | N | N | N | N | N | N | N | Y | Y | N | Y | N | Y | N | N |
| **19** | Y | Y | N | Y | N | Y | N | N | Y | Y | Y | Y | Y | N | Y | N | N | Y | N | Y | N |
| **20** | Y | Y | N | Y | Y | Y | Y | Y | Y | Y | Y | Y | Y | N | N | N | N | Y | Y | Y | N |
| **21** | Y | Y | Y | Y | N | Y | N | N | Y | N | Y | Y | N | N | N | N | N | Y | N | N | Y |
| **22** | Y | Y | N | Y | Y | N | Y | Y | N | Y | N | N | N | Y | Y | N | N | Y | Y | Y | Y |
| **23** | Y | Y | Y | Y | Y | Y | Y | Y | N | Y | Y | Y | Y | N | N | N | Y | Y | Y | Y | N |
| **24** | Y | Y | Y | Y | Y | Y | Y | Y | Y | Y | Y | Y | Y | Y | Y | N | Y | N | Y | Y | Y |
| **25** | Y | Y | Y | Y | Y | Y | N | N | Y | Y | Y | Y | Y | N | N | N | Y | N | Y | N | Y |
| **26** | Y | Y | Y | Y | Y | Y | Y | Y | Y | Y | Y | Y | Y | Y | Y | Y | Y | Y | N | Y | N |
| **27** | Y | Y | N | Y | Y | Y | Y | Y | Y | N | N | Y | Y | N | N | N | N | N | N | Y | N |
| **28** | Y | Y | Y | Y | Y | Y | Y | N | Y | Y | Y | Y | Y | Y | Y | N | Y | Y | Y | N | N |
| **29** | Y | Y | Y | Y | Y | N | Y | Y | Y | Y | Y | Y | Y | Y | Y | N | Y | N | Y | N | Y |
| **30** | Y | Y | Y | Y | Y | Y | Y | Y | Y | Y | Y | Y | Y | Y | Y | N | Y | N | Y | Y | N |
| **31** | U | U | U | U | U | U | U | U | U | U | U | U | U | U | U | U | U | U | U | U | U |
| **32** | U | U | U | U | U | U | U | U | U | U | U | U | U | U | U | U | U | U | U | U | U |

ANX: anxiety; AST: asthenia; BP: back pain; CHP: chest pain; CHT: chest tightness; CO: cough; CP: cervical pain; DIA: diarrhea; DIZ: dizziness; DYS: dyspnea; GD: general discomfort; JP: joint pain; LM: low mood; LGF: low grade fever; LOC: loss of concentration; MF: memory failure; MI: migraine; MP: muscle pain; N: no; PAL: palpitations; RAS: rashes; TIN: tingling in the extremities; U: unknown; Y: yes
