# Supplementary material for: Immune dysregulation and endothelial dysfunction associate with a pro-thrombotic profile in Long COVID
Source: Front Immunol. 2025 Oct 16;16:1613195. doi: 10.3389/fimmu.2025.1613195 (PMC12571756; doi:10.3389/fimmu.2025.1613195)
Supplement: Supplementary file 2 [file Table2.docx]

**Supplemental Table 2.** Sociodemographical and clinical characteristics of Recovered participants.

| **ID** | **Age (years)** | **Gender (M/F)** | **Time from clinical onset to sample (months)** | **Hospitalization due to acute COVID-19 (Y/N; days)** | **Comorbidities (Y/N)** | | | | | | **Current treatments (Y/N; which)** | **COVID-19 vaccines**  **(Y/N; which)** | **Doses of COVID-19 vaccines (no.)** | **Breakthrough infection**  **(Y/N; no.)** |
| --- | --- | --- | --- | --- | --- | --- | --- | --- | --- | --- | --- | --- | --- | --- |
|  |  |  |  |  | **DM** | **DL** | **HTA** | **TD** | **AID** | **ANX** |  |  |  |  |
| **1** | 66 | M | 24 | N | Y | Y | N | N | N | N | Y; CAR | Y; Pfizer/Pfizer/Pfizer | 3 | N |
| **2** | 60 | F | 24 | N | N | N | N | N | N | Y | Y; AD | Y; Pfizer/Pfizer/Pfizer | 3 | N |
| **3** | 52 | F | 24 | N | N | N | N | N | N | Y | Y; AD | Y; Pfizer/Pfizer/Pfizer | 3 | N |
| **4** | 55 | F | 24 | N | N | N | N | N | N | N | N | Y; Pfizer/Pfizer/Pfizer | 3 | N |
| **5** | U | M | 24 | N | N | N | N | N | N | N | N | Y; Pfizer | 1 | N |
| **6** | 28 | F | 24 | N | N | N | N | N | N | Y | Y; AD/AL | Y; Pfizer/Pfizer/Pfizer | 3 | N |
| **7** | 49 | F | 24 | N | N | N | Y | N | Y | N | Y; AD/CAR | Y; Pfizer/Pfizer/Pfizer | 3 | Y; 1 |
| **8** | 54 | F | 24 | N | N | N | N | N | N | N | N | Y; Pfizer/Pfizer/Pfizer | 3 | N |
| **9** | 59 | F | 24 | N | N | Y | N | N | N | Y | Y; AD/AL/CAR | Y; Pfizer/Pfizer/Pfizer | 3 | N |
| **10** | 31 | F | 24 | N | N | N | N | N | N | N | N | Y; Pfizer/Pfizer/Pfizer | 3 | Y; 1 |
| **11** | 29 | M | 24 | N | N | N | N | N | N | N | N | Y; Pfizer/Pfizer/Pfizer | 3 | N |
| **12** | 27 | F | 23 | N | N | N | N | N | N | N | Y; IM/VIT/AN | Y; Pfizer/Pfizer/Pfizer | 3 | Y; 1 |
| **13** | 52 | F | 24 | N | N | N | Y | Y | N | Y | Y; AD/AL/CAR | Y; Pfizer/Pfizer | 2 | N |
| **14** | 34 | M | 24 | N | N | N | N | N | N | N | N | Y; Pfizer/Pfizer/Pfizer | 3 | N |
| **15** | 30 | M | 24 | N | N | N | N | N | N | N | Y; IM | Y; Pfizer/Pfizer/Pfizer | 3 | N |
| **16** | 65 | F | 24 | N | N | N | Y | Y | Y | N | Y; CAR | Y; Pfizer/Pfizer/Pfizer | 3 | N |
| **17** | 50 | F | 24 | N | N | Y | N | N | N | N | Y; CAR | Y; Pfizer/Pfizer/Pfizer | 3 | Y; 1 |
| **18** | 40 | F | 24 | N | N | N | N | N | N | Y | Y; AD/AL | Y; Pfizer | 1 | U |
| **19** | 56 | F | 24 | N | N | Y | Y | N | N | N | Y; CAR | Y; Pfizer/Pfizer/Pfizer | 3 | N |
| **20** | 61 | M | 24 | N | N | N | N | U | N | U | U | Y; Pfizer/Pfizer/Pfizer | 3 | N |
| **21** | 52 | F | 24 | N | N | N | N | N | N | N | N | Y; Pfizer/Pfizer/Pfizer | 3 | N |
| **22** | 73 | F | U | N | N | Y | Y | U | N | U | U | U | U | U |
| **23** | 66 | F | 24 | N | N | Y | N | U | N | U | U | Y; AstraZeneca/ AstraZeneca / Moderna | 3 | N |
| **24** | U | M | 24 | N | N | Y | N | N | N | N | N | Y; Pfizer/Pfizer/Pfizer | 3 | U |
| **25** | 32 | F | 24 | N | N | N | N | N | N | N | N | Y; Pfizer/Pfizer | 2 | N |
| **26** | 61 | F | 24 | N | N | N | N | N | N | Y | Y; AL | U | U | U |
| **27** | 41 | F | 22 | N | N | N | N | N | N | N | N | Y; Pfizer/Pfizer/Pfizer | 3 | N |
| **28** | 46 | F | 24 | N | N | N | N | N | N | N | N | Y; Pfizer/Pfizer/Pfizer | 3 | Y; 1 |
| **29** | 50 | F | 24 | N | N | N | N | N | N | N | N | Y; Pfizer/Pfizer/Pfizer | 3 | N |
| **30** | 40 | M | 24 | N | N | N | N | N | N | N | Y; AN | Y; Pfizer/Pfizer/Pfizer | 3 | N |
| **31** | 56 | F | 24 | N | N | N | N | N | N | N | Y; AN | Y; Pfizer/Pfizer/Pfizer | 3 | N |
| **32** | 30 | M | 22 | N | N | N | N | N | N | N | N | Y; Pfizer/Pfizer/Pfizer | 3 | N |
| **33** | 50 | F | 24 | N | N | N | N | N | N | N | Y; IM | Y; Pfizer/Pfizer/Pfizer | 3 | N |
| **34** | 38 | F | 24 | N | N | N | Y | N | N | N | Y; AA/CAR | Y; Pfizer/Pfizer/Pfizer | 3 | N |
| **35** | 72 | M | U | N | N | N | Y | U | N | U | U | U | U | U |

AA: asthma and allergic rhinitis; AD: anti-depressants; AID: autoimmune disease; AL: anxiolytics; AN: analgesics/anti-inflammatories; ANX: anxiety; CAR: cardiovascular; DL: dyslipidemia; DM: diabetes mellitus; F: female; HTA: hypertension; IM: immunomodulators; M: male; N: no; TD: thyroid disorders; U: unknown; VIT: vitamins; Y: yes
